# Complete Genome Sequence of Lacticaseibacillus rhamnosus CAU 1365, Isolated from Kimchi

**DOI:** 10.1128/MRA.00932-21

**Published:** 2021-12-09

**Authors:** Yunjeong Lee, Jong-Hwa Kim, Wonyong Kim

**Affiliations:** a Department of Microbiology, Chung-Ang University College of Medicine, Seoul, Republic of Korea; University of Arizona

## Abstract

Here, we report the whole-genome sequence of Lacticaseibacillus rhamnosus CAU 1365, which was isolated from kimchi. The genome was composed of 1 contig with a total length of 2,991,039 bp and had 2,658 coding sequences, including 62 tRNA genes and 15 rRNA genes.

## ANNOUNCEMENT

The genus *Lacticaseibacillus* is a member of the family *Lactobacillaceae* within the class *Bacilli*. There are currently 26 species with validly published names listed in the DSMZ database (https://lpsn.dsmz.de/genus/lacticaseibacillus). Lactobacillus rhamnosus of the genus *Lactobacillus* has been reclassified as Lacticaseibacillus rhamnosus (1). L. rhamnosus is a Gram-positive, facultative, and heterofermentative lactic acid bacterium that can be found in various fermented dairy products, as well as the intestinal tract, vagina, and oral cavity. This species is important as a potential probiotic that exerts antimicrobial activity (2).

We isolated L. rhamnosus CAU 1365 from kimchi that had been purchased from the Malli local market (Seoul, Republic of Korea). The kimchi samples were serially diluted with 0.85% (wt/vol) peptone water and were spread on De Man-Rogosa-Sharpe (MRS) agar (BD Difco, Detroit, MI, USA). After incubation for 2 days at 37°C under aerobic conditions, a single colony was purified by repeated streaking on MRS plates. The isolate was characterized by PCR amplification of the 16S rRNA gene with the universal primers 8F (5′-AGAGTTTGATCCTGGCTCAG-3′) and 1525R (5′-ACGGCTACCTTGTTACGACTT-3′) (3). The purified gene was sequenced directly with an automated 3730 DNA sequencer (Applied Biosystems, Foster City, CA, USA), and the sequence similarity levels among closely related strains obtained from the GenBank database were calculated by using BLAST (https://blast.ncbi.nlm.nih.gov/Blast.cgi). Based on the results of 16S rRNA gene sequence analyses, L. rhamnosus CAU 1365 showed the greatest similarity to the L. rhamnosus type strain JCM 1136 (100%; GenBank accession no. BALT01000058).

**FIG 1:**
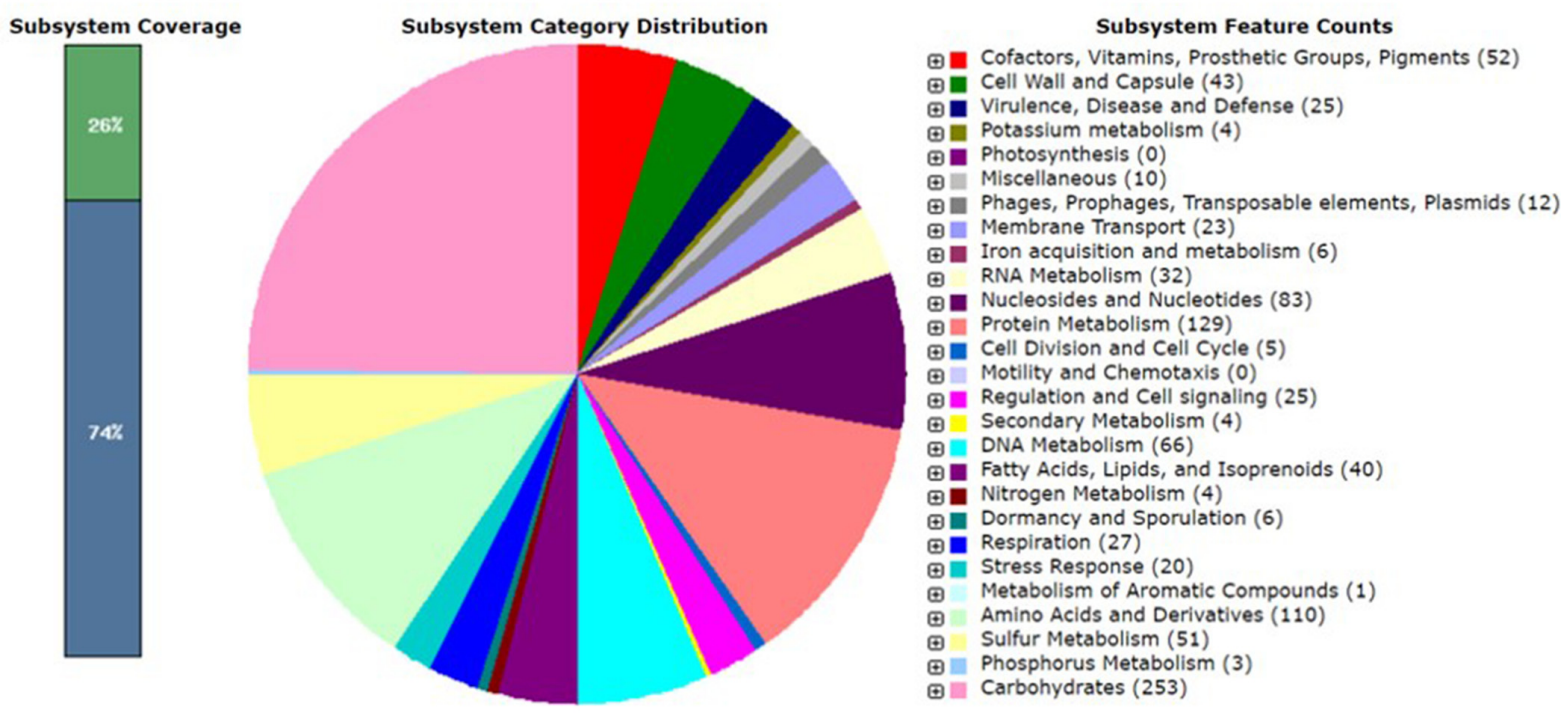
Functional category distribution for the genome of L. rhamnosus CAU 1365 according to RAST analysis.

Purified colonies were inoculated into MRS broth and cultivated overnight at 37°C before genomic DNA extraction. The genomic DNA of L. rhamnosus CAU 1365 was extracted using a genomic DNA extraction kit (iNtRON, Seongnam, Republic of Korea) according to the manufacturer’s instructions. The single Pacific Biosciences (PacBio) library was constructed with a SMRTbell template preparation kit (PacBio, Menlo Park, CA, USA); the library insert sizes were 15 to 20 kb, using the BluePippin size selection system (4). The library was sequenced using the RS II platform (PacBio) at Macrogen (Seoul, Republic of Korea), which generated 132,726 PacBio subreads with a mean subread length of 9,358 bp. The sequences were assembled *de novo* with RS Hierarchical Genome Assembly Process (HGAP) Assembly v. 3.0 using PacBio library preparation. *De novo* assembly consisted of assembly with PreAssembler v. 2.0, alignment by Basic Local Alignment with Successive Refinement (BLASR) v. 1.0, and assembly polishing with Quiver v. 1.0 in single-molecule real-time (SMRT) Portal v. 2.3 (5–7). The contig was identified as circular based on the presence of identical ends. Based on the results of BLASTN v. 2.7.1+ analysis, the assembly resulted in a single circular contig, which was a chromosome of 2,991,039 bp (coverage depth, 332×; G+C content, 46.8%). Genome annotation using Prokka v. 1.13 (8) and the NCBI Prokaryotic Genome Annotation Pipeline (PGAP) v. 5.3 (9) predicted 2,781 genes, of which 2,658 are protein-coding genes. A total of 80 RNAs were predicted, including 15 rRNAs (5S, 5 genes; 16S, 5 genes; 23S, 5 genes), 62 tRNAs, and 3 noncoding RNAs. In addition, the genome sequence was annotated by Rapid Annotations using Subsystems Technology (RAST) (10). According to the RAST results, the functional categories of protein metabolism (129 genes), amino acids and derivatives (110 genes), and carbohydrates (253 genes) were the predominant (>100 genes) subsystem categories for the genome (Fig. 1). Notably, there were lactose and galactose utilization-related and lactate fermentation-related gene functions. Default parameters were used for all software unless otherwise specified.

### Data availability.

The draft genome sequence is available in the DDBJ/ENA/GenBank databases under GenBank assembly accession no. GCA_019967935.1. The raw sequencing data were deposited in the Sequence Read Archive (SRA) database under accession no. SRR16018324.
